# Case Report: Novel endoscopic characteristics of early-stage autoimmune gastritis from two cases: the fog is lifting

**DOI:** 10.3389/fimmu.2025.1550919

**Published:** 2025-10-08

**Authors:** Yiming Song, Jianing Yan

**Affiliations:** ^1^ Department of Gastroenterology, the People’s Hospital of Fenghua, Ningbo, Zhejiang, China; ^2^ Department of Gastroenterology, the First Affiliated Hospital of Ningbo University, Ningbo, Zhejiang, China

**Keywords:** autoimmune gastritis, gastrin, clinical characteristic, endoscopic appearence, case report

## Abstract

With the development of research, the clinical characteristics of autoimmune gastritis (AIG) have become much clearer. However, due to its occult endoscopic appearance, it remains difficult to diagnose early, including ultra-early AIG. More cases and typical clinical characteristics should be proposed and concluded to guide clinicians. Hence, we selected two novel cases of early AIG and summarized their common endoscopic features in order to improve diagnostic ability. We found two interesting features for early AIG: 1) deep reticular blood vessels in the lower part of the cardia mucosa and 2) various manifestations of crypt opening shown on narrow-band imaging (NBI) magnification, such as dilation, pinhole-like, shrinkage, and disappearance. Future studies promise even more progress with the hope that these features can be found in more cases.

## 1 Introduction

In general, gastritis is classified into autoimmune gastritis (AIG, type A) and bacterial gastritis (type B). With increasing attention being paid to the relationship between *Helicobacter pylori* and gastric cancer, the pathogenic mechanisms and disease evolution of type B gastritis are reasonably well established. AIG is characterized by the disappearance of the oxyntic mucosa associated with hypochlorhydria, causing a reduced or absent secretion of gastric acid, accompanied by higher levels of anti-parietal cell antibodies and intrinsic factor antibodies (1). Although typical endoscopic characteristics such as reverse atrophy, hyperplastic polyps, and sticky adherent dense mucus help in the diagnosis of AIG, AIG with high remnant oxyntic mucosa remains difficult to identify (2). Hence, there is an urgent need to determine novel endoscopic characteristics and construct a more complete system for the diagnosis of AIG.

In this study, we report on two cases of early AIG and point out that a grid-like microvascular mesh in the lesser curvature of the gastric body under the cardia, an irregular crypt opening (CO), can be a novel indicator of early AIG. CO indicates the structure of the gastric mucosal glands opening at the bottom of the pit. In the fundic gland area, the gastric pits are shallow and the glands are closely arranged, vertically, and not distorted. The openings of the gastric crypts are round or oval. Under magnifying endoscopy, the subepithelial capillary network was shown to be surrounded by the light brown edge of the crypt epithelium, and the central dark streaks were faintly visible. We believe that our efforts will shed new light on the identification of early AIG to increase the clinical discovery rates.

## 2 Case 1

A 52-year-old Chinese woman underwent upper gastrointestinal endoscopy for a health examination at our hospital. She had a history of Hashimoto’s thyroiditis and impaired thyroid function. She had no history of *H. pylori* or long-term medication use. Her family history was unremarkable.

**Figure 1:**
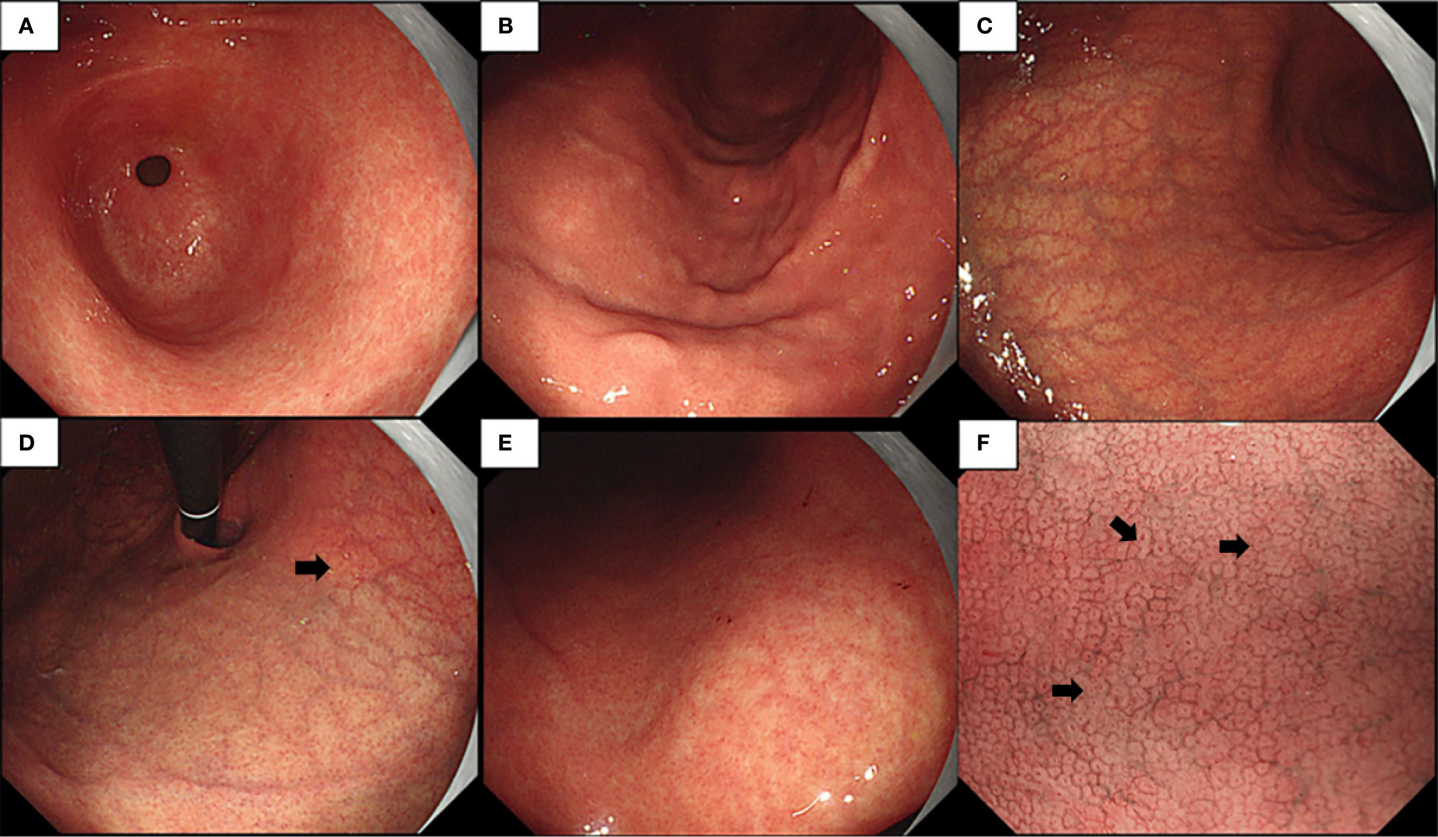
**(A–E)** Endoscopic examination observed a normal gastric antral mucosa **(A)**, smooth mucosa of greater curvature of the corpus with decreasing fold **(B)**, limited reverse atrophy **(C)**, deep reticular blood vessels in the lower part of the cardia mucosa **(D)**, and fusion of white atrophy foci at the lower part **(E)**. **(F)** Narrow-band imaging (NBI) magnification showing various manifestations of crypt opening (CO), such as dilation, pinhole-like, shrinkage, and disappearance.

Endoscopic examination revealed a normal antral mucosa (**Figure 1A**) and a smooth mucosa of the greater curvature of the corpus with a decreasing fold (**Figure 1B**), while circumscribed reverse atrophy was found in an inflated state. After full inflation, a grid-like microvessel was found in the upper part of the lesser curvature of the corpus under the cardia (**Figure 1C**), while fusion of white atrophic foci was observed in the lower part (**Figures 1D, E**). Moreover, the CO became linear, smaller, and then disappeared (**Figure 1F**) on narrow-band imaging (NBI) combined with magnifying endoscopy. Biopsy was performed at the atrophic foci and antral mucosa.

**Figure 2:**
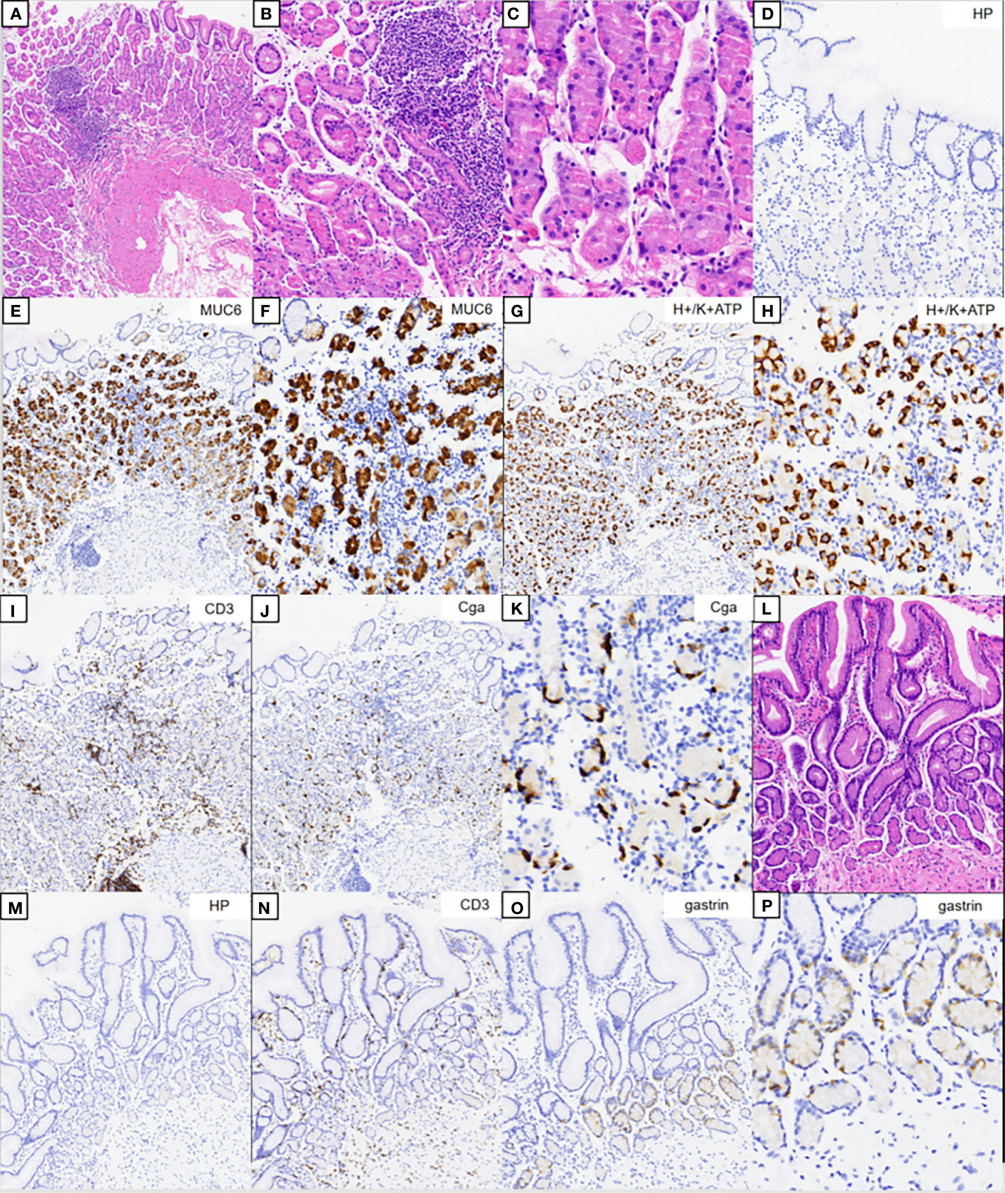
Pathology results. **(A–C)** Hematoxylin–eosin (HE) staining results of the corpus. **(B, C)** Magnification of the parietal and chief cells. **(D)** Immunohistochemical staining of *Helicobacter pylori*. **(E, F)** Results of MUC6 staining. **(G, H)** Results of H/K-ATPase staining. **(I)** Results of CD3 staining. **(J, K)** Results of CgA staining. **(L)** HE staining results of the gastric antrum. **(M)** Immunohistochemical staining of *H. pylori*. **(N)** Results of CD3 staining. **(O, P)** Results for gastrin.

The hematoxylin–eosin (HE) staining results of the corpus are shown in **Figures 2A–C**. No significant atrophy of the gastric corpus glands was observed. In contrast, there was extensive infiltration of inflammatory cells in the lamina propria (**Figure 2A**), with swelling and degeneration of parietal cells (**Figure 2B**). A weaker alkalophilic preference was observed in the chief cells (**Figure 2C**). *H. pylori* was not detected in the *H. pylori* staining (**Figure 2D**). The MUC6 staining results are shown in **Figures 2E, F**. H/K-ATPase staining showed unclear differentiation between the chief and parietal cells (**Figures 2G, H**). Chromogranin A (CgA) staining showed linear proliferation of neuroendocrine cells (**Figures 2J, K**). CD3 staining showed lymphocyte infiltration in the lamina propria of the corpus (**Figure 2I**), but not in the gastric antrum (**Figure 2N**). The results of the HE staining of the gastric antrum are shown in **Figures 2L–P**. Gastrin staining revealed the proliferation of G cells in the gastric antrum (**Figures 2O, P**). *H. pylori* was not detected in the *H. pylori* staining (**Figures 2D, M**).

The serological test resulted positive for anti-parietal cell antibodies and anti-thyroid peroxidase antibodies, but negative for anti-intrinsic factor antibodies. The routine blood test results and the anemia indicators were normal.

Early-stage AIG is diagnosed based on the following features: 1) endoscopy: circumscribed reverse atrophy; grid-like microvessels in the upper part of the lesser curvature of the corpus under the cardia; and linear or smaller appearance or disappearance of the CO; 2) histopathology: parietal cell degeneration, neuroendocrine cell proliferation, and absence of *H. pylori*; 3) serology: parietal cell antibody (PCA) positivity; and 4) medical history: Hashimoto’s thyroiditis.

## 3 Case 2

A 30-year-old Chinese woman underwent upper gastrointestinal endoscopy for recurrent abdominal pain. She had a history of *H. pylori* eradication, as confirmed by the C14 urea breath test, and Hashimoto’s thyroiditis with impaired thyroid function. Her mother also had Hashimoto’s thyroiditis.

**Figure 3:**
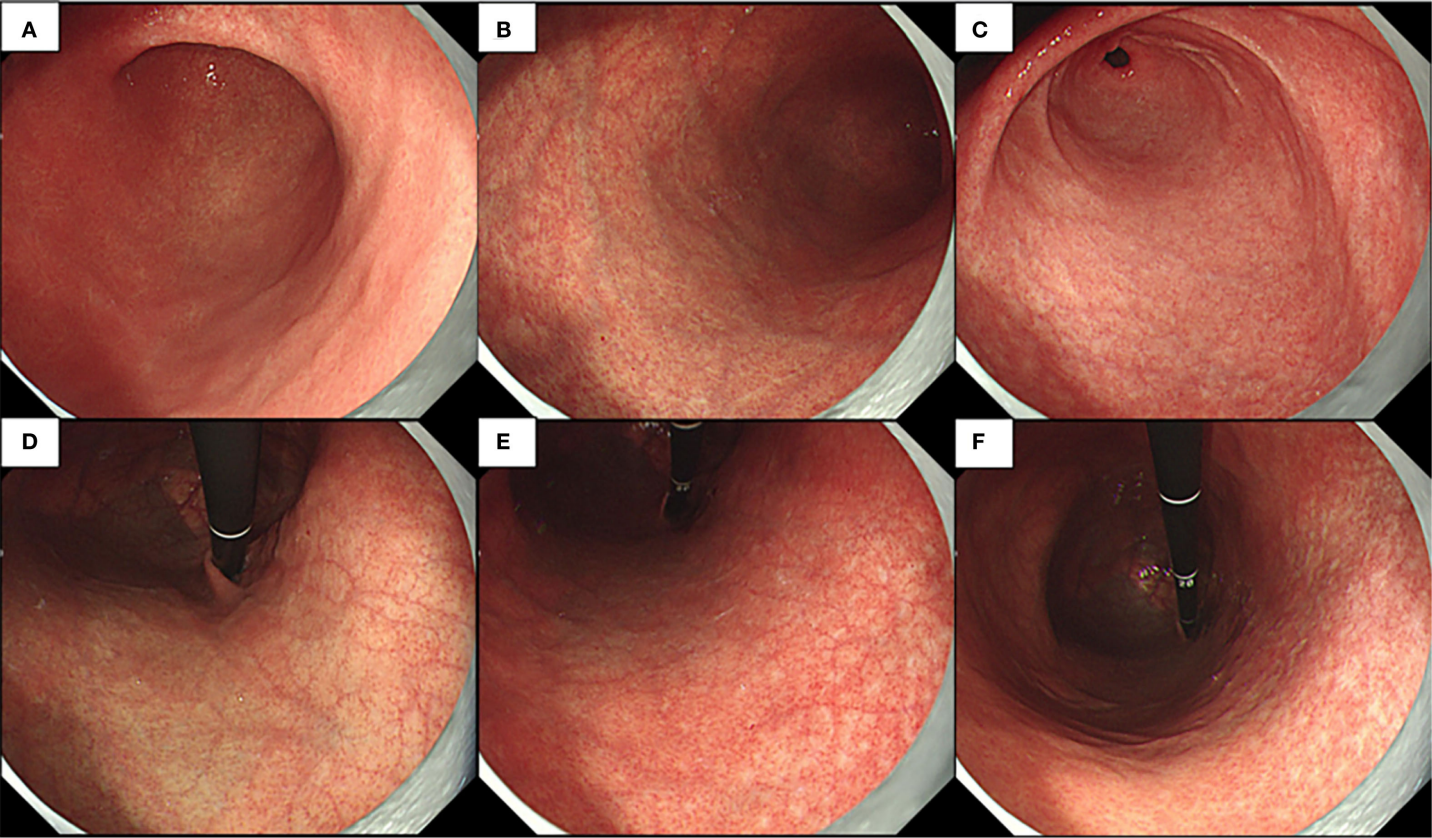
Endoscopic examination observed normal mucosa of greater curvature of the lower part of the gastric body and little atrophy in the gastric angle **(A)**, mild atrophy at greater curvature of the upper part of the gastric body **(B)**, little atrophy in the gastric antral mucosa **(C)**, deep reticular blood vessels in the lower part of the cardia mucosa **(D)**, transition band of grid-like vessels and atrophy foci at the middle part of the lesser curvature of the gastric body **(E)**, and a variety of atrophy foci that could be discovered at the lower part **(F)**.

Endoscopic examination revealed a normal mucosa in the greater curvature of the lower part of the gastric body (**Figure 3A**). Mild atrophy was observed in the greater curvature of the upper part of the gastric body (**Figure 3B**). Atrophy was also observed at the gastric antral stage, consistent with appearance after *H. pylori* eradication (**Figure 3C**). After full inflation, grid-like microvessels were observed in the upper part of the lesser curvature of the corpus under the cardia (**Figure 3D**). The transition band of the grid-like vessels and atrophic foci was located in the middle of the lesser curvature of the gastric body (**Figure 3E**). Various atrophic foci were discovered in the lower part due to previous infection with *H. pylori* (**Figure 3F**).

**Figure 4:**
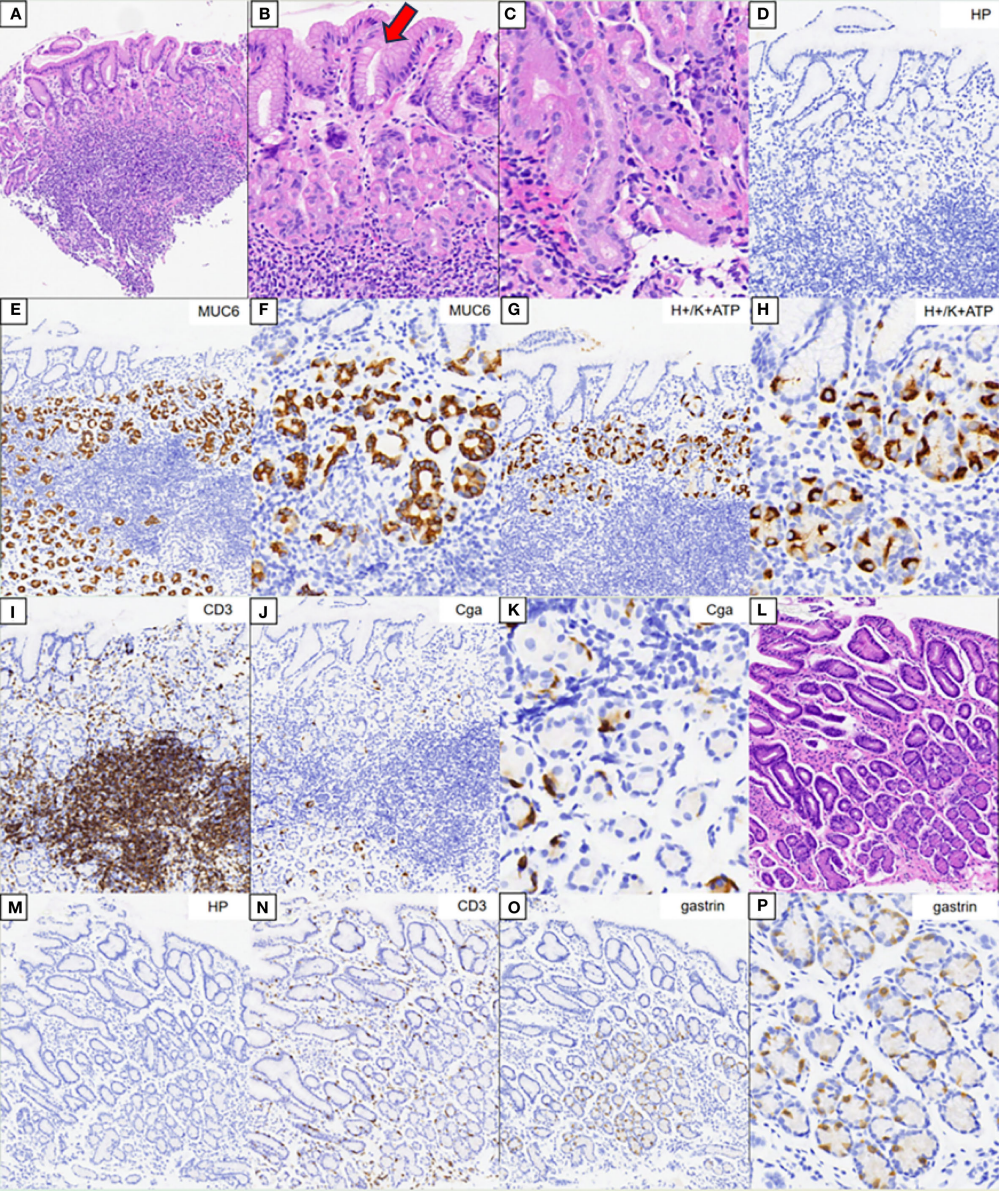
Pathology results. **(A–C)** Hematoxylin–eosin (HE) staining results of the corpus. **(B, C)** Magnification of the parietal and chief cells. **(D)** Immunohistochemical staining of *Helicobacter pylori*. **(E, F)** Results of MUC6 staining. **(G, H)** Results of H/K-ATPase staining. **(I)** Results of CD3 staining. **(J, K)** Results of CgA staining. **(L)** HE staining results of the gastric antrum. **(M)** Immunohistochemical staining of *H. pylori*. **(N)** Results of CD3 staining. **(O, P)** Results for gastrin.

The HE staining results of the gastric body are shown in **Figures 4A–C**. There was extensive infiltration of inflammatory cells in the lamina propria of the gastric body without atrophy. A weaker alkalophilic preference was present in the chief cells, and distinction between the chief cells and parietal cells was not clear. The MUC6 staining results are shown in **Figures 4E, F**. The H/K-ATPase and CD3 staining results are shown in **Figures 4G–I**. CgA staining did not show proliferation of neuroendocrine cells (**Figures 4J, K**). The HE staining results of the gastric antrum are shown in **Figure 4L**. The CD3 staining results are shown in **Figure 4N**. Gastrin staining revealed the proliferation of some G cells in the gastric antrum (**Figures 4O, P**). *H. pylori* was not detected in the *H. pylori* staining (**Figures 4D, M**).

The serological test resulted positive for anti-parietal cell antibodies and anti-thyroid peroxidase antibodies. The routine blood test results and the anemia indicators were normal.

In summary, this patient was diagnosed with early-stage AIG based on the following features: 1) endoscopy: grid-like microvessels in the upper part of the lesser curvature of the corpus under the cardia; 2) histopathology: unclear differentiation between chief and parietal cells, G-cell proliferation, inflammatory cell infiltration, and absence of *H. pylori*; 3) serology: PCA positivity; and 4) medical history: Hashimoto’s thyroiditis.

## 4 Discussion

The diagnostic criteria for AIG are based on the following: 1) typical endoscopic or pathological findings and 2) positive results for anti-parietal cell or anti-intrinsic factor antibodies. The pathological features of early AIG include the following:

1. Normal bilayer structure of the fundic gland, parietal/mucinous neck cell layer, and blurred principal cell layer, but the parietal and mucinous neck cell layers are retained.
2. The majority of parietal cells are still present, although swelling (swollen = pseudohypertrophy) with lumen protrusion and shedding can be observed.
3. Chief cells are often transformed into pyloric gland cells or mucous neck cells (pseudopyloric gland cells).
4. Enterochromaffin-like (ECL) cells can mildly proliferate, with mild-to-moderate lymphocyte/plasma cell infiltration in the gastric glands.
5. G cells mildly proliferate in the pyloric gland mucosa (3, 4).

Both patients were diagnosed with early-stage AIG based on these characteristics, and we can comprehensively understand the clinical diagnostic value by reviewing the endoscopic features.

In this study, we present two cases of early AIG without the characteristic features of gastric pit swelling and remnant oxyntic mucosa mentioned in the literature. In the first case, limited reverse atrophy was observed on endoscopy, and deep reticular blood vessels were visible in the lower part of the cardia mucosa. NBI magnification revealed various manifestations of the CO, such as dilation, pinhole-like appearance, shrinkage, and disappearance. We found that the deep reticular blood vessels in the lower part of the cardia mucosa were due to the infiltration of deep inflammatory cells in AIG, combined with the reduction of parietal and chief cells in the lamina propria of the mucosa, leading to the thinning of the gastric wall (atrophic changes in the cardia mucosa area). Therefore, under sufficient inflation, deep blood vessels were observed. With disease progression, these deep blood vessels will become increasingly obvious, and the atrophy range will become much larger, eventually showing the typical endoscopic manifestations of advanced AIG (5).

We analyzed the various manifestations of the CO under magnifying endoscopy and found that they were due to the nourishment of the gastric pit epithelium by gastrin, leading to the excessive formation and maturation of glandular fossa epithelial cells at the edge of the glandular fossa. Moreover, as the inflammation in early AIG occurs in the deep lamina propria of the mucosa with less surface inflammation, the lamina propria of the mucosal glands is still relatively preserved. The arrangement of the glandular fossa epithelial layer with hyperplasia remained, but there were morphological differences, resulting in different depths of the glandular fossa openings (6). Thus, various manifestations of the CO were observed under endoscopy. With further progression of the disease, the glandular fossa epithelium gradually proliferates, and the glandular fossa openings gradually become shallower, resulting in the inability to observe the CO, eventually showing signs of desquamation.

Let us now discuss the second case. This patient also had deep reticular blood vessels in the lower part of the cardia mucosa; a special feature was that this is a post-eradication case. The evidence for post-eradication included the following: 1) The patient had a high value in the C14 urea breath test 2 years ago, and this breath value became negative after regular eradication treatment (self-reported). 2) The overall background mucosa under endoscopy was smooth, with a Kimura–Takemoto classification of C-2 with punctate atrophy, which was consistent with the post-eradication manifestation. It was found that, whether in *H. pylori*-negative stomach or in a post-eradication background, both patients had deep reticular blood vessels in the lower part of the cardia mucosa, suggesting the specificity of this feature. One important reason for choosing this case was that the atrophy range caused by *H. pylori* was small and the AIG was in the early stage. In the middle of the lesser curvature of the gastric body, the intersection between the middle and the upper reticular blood vessels and punctate atrophy foci were observed, indicating collision of the two atrophy patterns. If *H. pylori* infection or further progression of AIG leads to the intersection and superposition of the two atrophy patterns, it would be impossible to diagnose the post-eradication background using endoscopy, causing great difficulties in the diagnosis of AIG (7).

## 5 Conclusion

Based on these two cases and references, we concluded on the following endoscopic features of early AIG: 1) deep reticular blood vessels in the lower part of the cardia mucosa and 2) NBI magnification revealing various manifestations of the CO, such as dilation, pinhole-like appearance, shrinkage, and disappearance. Pathological and serological results are necessary for any patient suspected of having early AIG.

## Data Availability

The original contributions presented in the study are included in the article/supplementary material. Further inquiries can be directed to the corresponding author.
